# Ethnopharmacology, Phytochemistry, and Pharmacological Properties of *Thymus satureioides* Coss.

**DOI:** 10.1155/2021/6673838

**Published:** 2021-01-27

**Authors:** Naoufal El Hachlafi, Abderrahim Chebat, Kawtar Fikri-Benbrahim

**Affiliations:** ^1^Microbial Biotechnology and Bioactive Molecules Laboratory, Sciences and Technologies Faculty, Sidi Mohamed Ben Abdellah University, P.O. Box 2202, Imouzzer Road, Fez, Morocco; ^2^Moroccan Anti Poison and Pharmacovigilance Center, P.O. Box 6671, Rabat, Morocco

## Abstract

*Thymus satureioides* Coss. (Lamiaceae) is a Moroccan medicinal plant locally known as “Azkouni” or “Zaitra.” It is widely used in traditional medicine to treat various ailments, including hypertension, diabetes, cold, fever, dermatological and circulatory disorders, immune problems, bronchitis, nociception, cooling, pharyngitis, cough, and influenza. The current review aims to critically summarize the literature on ethnopharmacological uses, chemical profile, and pharmacological investigations of *T. satureioides* in order to provide data support and scientific evidences for further investigations. Electronic databases such as Scopus, PubMed, Web of Science, SciFinder, ScienceDirect, Google Scholar, and Medline were used to gather data on *T. satureioides*. Chemical characterization of *T. satureioides* essential oils (EOs) and extracts allowed to identify a total of 139 bioactive compounds, mainly belonging to the terpenoids, phenolic acids, and flavonoids classes. *T. satureioides* especially its essential oils exhibited numerous biological activities such as antibacterial, antifungal, anti-inflammatory, antioxidant, antidiabetic, anticancer, antiparasitic, and hypolipedemic activities. In light of these findings, further studies to transmute the traditional application of *T. satureioides* into scientific-based information are strongly required. Additional *in vivo* pharmacological studies are recommended to validate the results of the *in vitro* studies. Moreover, comprehensive preclinical and clinical trials on the pharmacological mechanisms of action of this plant and its bioactive compounds on molecular targets should be performed. Finally, more efforts must be focused on toxicological assessments and pharmacokinetic studies, in order to ensure the safety and the efficiency of *T. satureioides*.

## 1. Introduction

*Thymus satureioides* Coss. is a perennial shrub (10–60 cm in height) belonging to the Lamiaceae family and the genus *Thymus* [1, 2]. *T. satureioides* is an endemic Moroccan medicinal plant locally known as “Azkuni” or “Zaitra” [3]. This species is widely distributed in the arid and semiarid habitats of the Moroccan High Atlas and Anti-Atlas [1, 4].

In Morocco, *T. satureioides* has been extensively used in folk medicine against numerous diseases, including arterial hypertension, diabetes, cold, fever [5, 6], dermatological and immune problems, digestive ailments [1, 7, 8], and metabolic disorders [9]. Ethnopharmacological investigations showed that *T. satureioides* is used for the treatments of bronchitis, skin ailments, nociception, circulatory disorders, urogenital problems, nervous and visual ailments, cooling, pharyngitis, cough, influenza, and as an antispasmodic agent [5, 10–12]. Phytochemical analysis of *T. satureioides* essential oils and extracts enabled to identify numerous bioactive compounds belonging to several chemical classes, including terpenoids, phenolic acids, flavonoids, steroids, alkaloids, and saponins [13–16].

Several pharmacological reports based on *in vitro* and *in vivo* studies have demonstrated that *T. satureioides*, especially its EOs, exhibit various biological activities such as antibacterial [17, 18], antifungal [19, 20], antioxidant [21, 22], antidiabetic [2], anticancer [23], anti-inflammatory [24], insecticidal [25, 26], and hypolipedemic effects [27]. However, the targeted mechanisms of these pharmacological properties have been poorly investigated.

Although numerous studies reported the ethnomedicinal properties and pharmacological effects of *T. satureioides*, to the best of our knowledge, no review was published to summarize these reports and suggest the future pharmacological applications of this plant. Therefore, this review was designed to critically summarize all published works on ethnomedicinal uses, phytochemistry, and pharmacological properties of *T. satureioides*. The current paper aims to provide data support and prospect concerning future research studies on the biological potential of *T. satureioides*.

## 2. Research Methodology

All published works about the ethnomedicinal uses, phytochemical composition, and biological activities of *T. satureioides* were collected, examined, and reported in the present review. An extensive bibliometric survey from different scientific databases such as ScienceDirect, PubMed, Scopus, Web of Science, SpringerLink, Google Scholar, and Medline was used to extract all relevant papers. A total of 79 peer-reviewed papers published in English and French languages were selected to compose this review. The data provided in case reports, editorial/letters, patents, conference papers, and symposiums were excluded because they were considered scientifically unreliable. The search keywords used are “*T. satureioides*, phytochemical composition of *T. satureioides, T. satureioides* EOs, biological activities of *T. satureioides*, the antimicrobial activity of *T. satureioides*, ethnobotanical study of *T. satureioides*, and the antioxidant effect of *T. satureioides*”. ChemDraw Ultra 12.0 Software was used to draw the chemical structures. IUPAC names of the reported chemical compounds were cheeked using PubChem databases (pubchem.ncbi.nlm.nih.gov).

## 3. Results and Discussion

### 3.1. Botany, Ecology, and Biogeographic Distribution

**Figure 1:**
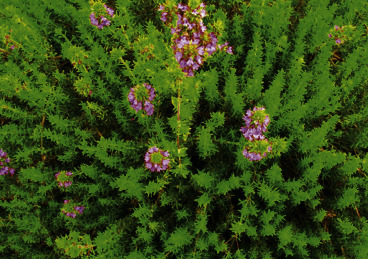
*Thymus satureioides* at flowering stage.

*T. satureioides* is a bushy perennial shrub (10–60 cm in height) with erect branches [1, 2] (Figure 1). Its leaves are opposite, linear, or lanceolate, curled at the edges, grayish on top, and tomentose at the base. The flowers are grouped into ovoid glomerules. The corolla is bilabiate (1/2 cm) with pink or whitish petals [3]. Reproduction of *T. satureioides* occurs via sexual (seeds) and asexual route through bursts of stump, cuttings, and marcottage [1].

*T. satureioides* is an endemic Moroccan plant, geographically found in the Mediterranean, Thermomediterranean, and Mesomediterranean series, in forest clearings, scrub, matorrals, and low and medium mountains up to 2200 m altitude [3, 27]. This species grows on siliceous limestone substratum and rocky to moderately earthy soils in the High Atlas and Anti-Atlas of Morocco. From a climatic point of view, *T. satureioides* is located in the arid to subhumid bioclimate, with hot, temperate, and fresh variants [3].

### 3.2. Ethnomedicinal Use

*T. satureioides* is one of the medicinal plants commonly used in Moroccan folk medicine to treat many pathological disorders, including diabetes, arterial hypertension, digestives ailments, cold, fever, and respiratory problems [5, 6].

Several ethnobotanical and ethnopharmacological surveys reported these practices and showed that the medicinal use of *T. satureioides* depends on the plant's part used (Table 1). The aerial parts of *T. satureioides* were used as a decoction and infusion to treat gastric disorders, chills, cold, fever, and headaches [11], as well as arterial hypertension and diabetes [5, 28]. In addition, Mouhajir et al. [35] showed that the aerial part decoction is used as food disinfectant and against cold and colic.

The whole plant is used to treat dermatological disorders, immune problems, digestive ailments, intestinal troubles, colds, and coughs [7, 8, 29]. The leaves of *T. satureioides* are mainly known to be used against metabolic disorders, in particular diabetes [9, 12, 31], as well as for the treatments of bloating and diarrhea [32] or against cooling, pharyngitis, cough, and influenza [10].

Other ethnomedicinal studies reported that *T. satureioides* was also used as an antispasmodic and antinociceptive agent, and for the treatment of bronchitis, skin ailments, circulatory disorders, urogenital problems, nervous and visual ailments, and menstruation pains [1, 5, 11, 12, 33].

### 3.3. Phytochemistry

Phytochemical screening of *T. satureioides* EOs and extracts revealed the presence of a total of 139 bioactive compounds, which can be grouped into three main chemical classes, including terpenoids, phenolic acids, and flavonoids (Table 2).

#### 3.3.1. Phenolic Compounds

Thanks to their phenolic group, the phenolic compounds such as phenolic acids, flavonoids, tocopherols, and tannins are considered as an important group of bioactive compounds that are responsible for a wide range of biological properties such as antimicrobial [51, 52], antioxidant [53] anticancer [54], and litholytic activities [55]. Besides their pharmacological potential, the phenolic compounds, particularly flavonoids, are involved in many physiological processes; they are included in the regulation and protection of vascular plants against several biotic and abiotic stresses [56–58].

There are few studies investigating the chemical composition of *T. satureioides* extracts. In fact, the phenolic profile of *T. satureioides* remains not well identified.

Khouya et al. [24] have examined the phenolic composition of the *T. satureioides* aqueous extracts and reported that they contain high levels of phenolic compounds, which are represented by rosmarinic acid as major phenolic acid and luteolin-7-glycoside and hesperetin as major flavonoids. Another study showed that *T. satureioides* aqueous extracts were rich in total polyphenols (456.73 ± 6.94 mg caffeic acid equivalent/g of dry plant) and in flavonoid group (172.79 ± 2.12 mg rutin equivalent/g of dry plant) with rosmarinic acid, hesperetin, and luteolin-7-glucoside as major phenolic compounds [13].

In a recent study, Tebaa et al. [59] showed that the aqueous extracts of *T. satureioides* aerial parts are rich in total polyphenols (285 ± 34.82 *μ*g gallic acid equivalent/mL aqueous extract), in total flavonoids (25.83 ± 4 *μ*g catechin equivalent/mL aqueous extract) and in total tannins (0.032 ± 0.002 *μ*g tannic acid equivalents/mL aqueous extract).

In addition, the chemical composition of the methanol extracts of *T. satureioides* analyzed by a combination of chromatographic tools (reverse-phase HPLC and ^1^H NMR analyses) revealed the presence of flavonoids as the main constituents with five essential compounds: luteolin-3′-O-glucuronide, luteolin-7-O-glucoside, eriodictyol-7-O-glucoside, aglycone luteolin, and thymonin [14]. However, other molecules such as ursolic acid and oleanolic acids were identified in the chloroform extract of *T. satureioides* [14].

The qualitative phytochemical analysis of *T. satureioides* extracts (hydromethanol, chloroform, ethyl acetate, and butanol extracts) enabled to detect the presence of flavonoids, catechols, gallic tannins, and anthraquinones [60]. Moreover, the quantitative HPLC analysis of crude and organic extracts of *T. satureioides* aerial parts showed the presence of phenolic acids (caffeic acid and rosmarinic acid) and the flavonoids quercetin and hesperetin in crud and methanolic extracts, whereas rosmarinic acid, hyperoside, quercetin, and hesperetin were detected in ethyl acetate extracts [39].

Interestingly, Kouar et al. [38] have determined the phytochemical profile of alcoholic extract of *T. satureioides* leaves, using the electrocoagulation and solvent extraction assays, and detected the presence of saponins, sterols, triterpene, tannins, and flavone aglycones. The quantitative analysis showed that *T. satureioides* alcoholic extract contains high levels of total polyphenols (70.2 ± 0.4 mg of gallic acid equivalents/g extract) and total flavonoids (52.7 ± 0.01 mg of quercetin equivalents/g extract). The high performance liquid chromatography (HPLC) analysis allowed to identify six compounds in this alcoholic extract, including four phenolic acids (**12–14, 16)** and two flavonoid compounds (**1–2**) [39].

**Figure 2:**
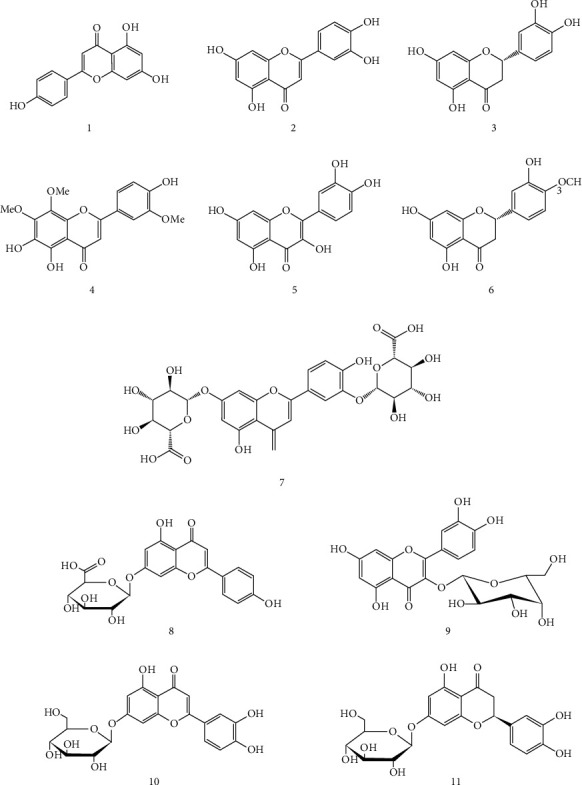
Flavonoid compounds isolated from *T. satureioides* extracts.

**Figure 3:**
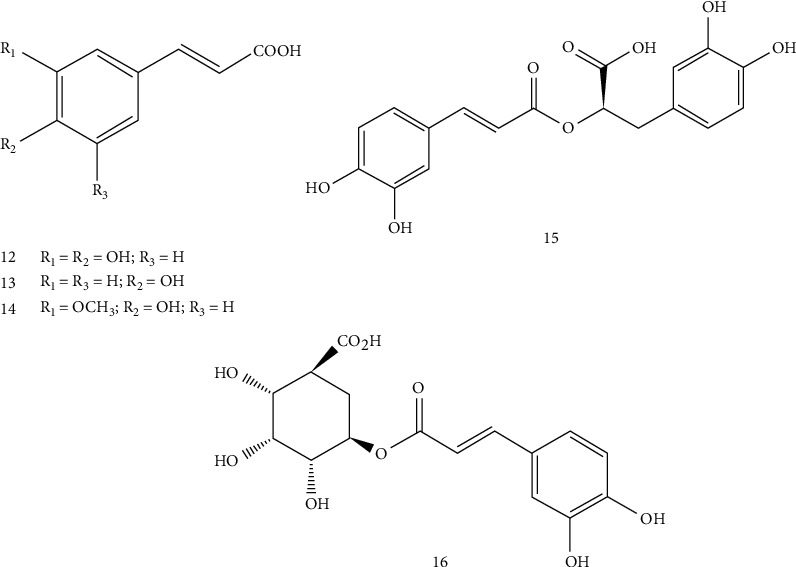
Phenolic acids identified in *T. satureioides.*

The phenolic compound content and nature vary depending on the extraction solvent, plant's part used, plant's origin, storage conditions, and analytical method used. Indeed, flavonoids are the main phenolic group detected in *T. satureioides* extracts with 11 compounds (**1–11**) (Figure 2). Moreover, five phenolic acids were identified (**12–16**) (Figure 3).

#### 3.3.2. Volatile Compounds

Numerous studies have investigated and characterized the chemical composition of *T. satureioides* EOs, particularly from the aerial parts.

**Figure 4:**
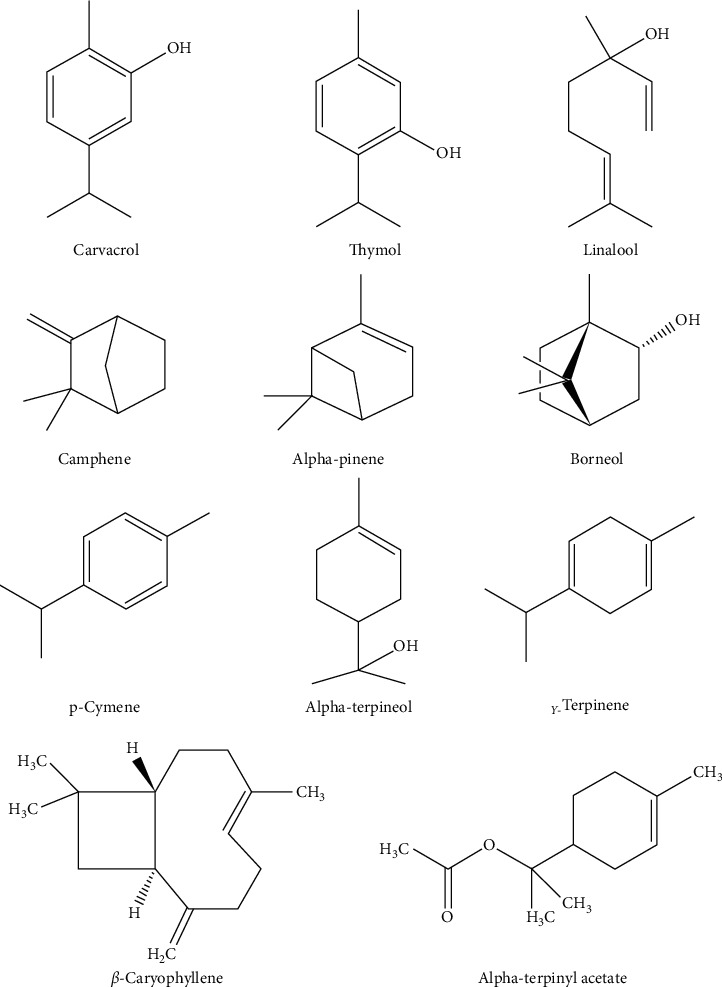
Chemical structure of the main terpenoids identified in *T. satureioides*.

The chemical analysis showed that *T. satureioides* EOs are mainly composed of borneol, thymol, carvacrol, camphene, *α*-pinene, *α*-terpineol, *p*-cymene, and linalool (Figure 4).

The percentages and the nature of these volatile compounds vary noticeably depending on several intrinsic and extrinsic factors of the plant, including geographical origin, phenological stage, genotype, plant's part used, and storage and extraction conditions [61, 62].

Sbayou et al. [43] indicated that borneol and thymol are the chief components of *T. satureioides* EOs with 26.45% and 11.24%, respectively, followed by *α*-terpinyl acetate (10.99%), *β*-caryophyllene (8.24%), and camphene (7.16%). The studies carried out on *T. satureioides* from the High Atlas of Morocco indicated that carvacrol (26.5%) and borneol (20.1%) are the main compounds of its EOs, while thymol was not identified [15, 49].

It is well seen that borneol, carvacrol, and thymol constitute the major proportion of the volatile compounds of *T. satureioides*. Indeed, in an earlier study, Jaafari et al. [23] described the EOs of *T. satureioides* harvested in Tiznit region as a “borneol chemotype (59.37%),” those of Marrakech region (Asni-My Brahim) as “carvacrol (35.90%) and borneol (30%) chemotypes,” and the one of Beni Mellal region (Bin El Widane) as “borneol (51.98%) and thymol (26.81%) chemotypes,” thus showing a variation in chemotypes of the *T. satureioides* EOs according to harvest zones.

A comparative study of *T. satureioides* leaf and flower EOs, using simultaneous GC-FID and GC-MS tools, showed major differences regarding the main compounds of these two plants' part EOs. Thereby, borneol (a monoterpene alcohol) was the main compound of the flowers EOs with 19.3%, followed by carvacrol (10.0%) and thymol (3.8%), while carvacrol (37%), thymol (13.7%), *γ*-terpinene (8.4%), and (E)-*β*-caryophyllene (6.6%) were the main components of the leaves EOs [41].

Another study revealed the presence of 68 volatile compounds representing 93.3% of *T. satureioides* aerial parts' total EOs using capillary gas chromatography and gas chromatography coupled to mass spectrometry (GC-MS) [40]. These volatile compounds mainly belong to the monoterpenoides class (monoterpene hydrocarbons, oxygenated monoterpenes, and phenolic monoterpenes) such as borneol, carvacrol, thymol, camphene, linalool, and camphor.

### 3.4.  Pharmacological Properties

**Figure 5:**
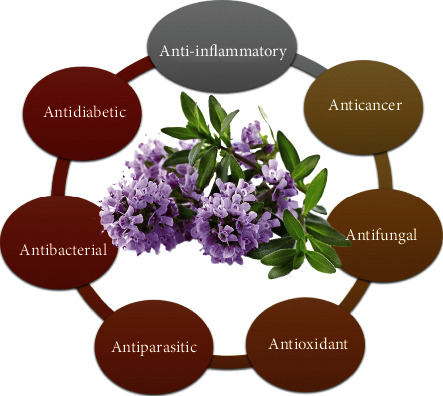
Pharmacological properties of *T. satureioides*.

Numerous pharmacological investigations have shown that *T. satureioides* essential oils and extracts obtained from different plant parts possess various biological activities, including antibacterial, antioxidant, antifungal, antiparasitic, anticancer, antidiabetic, and anti-inflammatory effects (Figure 5).

#### 3.4.1. Antibacterial Activity

The antibacterial activity of *T. satureioides* EOs and extracts, against a panel of bacterial strains, including Gram-positive and Gram-negative bacteria, was reported in the literature [16, 63, 64].

Indeed, the EOs obtained from different parts of *T. satureioides* were evaluated against several pathogenic bacteria known by their drug multiresistance, including *Pseudomonas aeruginosa*, *Klebsiella pneumoniae*, *Escherichia coli*, *Acinetobacter baumannii*, and methicillin-resistant *Staphylococcus aureus* (MRSA).  Table 3 summarizes the published works that investigated the antibacterial activities of *T. satureioides*. It lists the parts used, the tested extracts, the methods used, the tested strains, and the main results obtained.

Ou-Yahia et al. [67] assessed the antibacterial effect of the *T. satureioides* aerial part EOs against *E. coli*, *Bacillus cereus*, *P. aeruginosa*, *Salmonella typhimurium*, *S. aureus*, *Micrococcus luteus*, and *Bacillus subtilis* and showed a variable antibacterial activity of the tested EOs. The highest activity was observed against *B. cereus* and *S. aureus* with MIC values of 0.015% and 0.03%, respectively, while *P. aeruginosa* was the most resistant bacterium with a MIC value of 1%.

In another study, testing of the bacteriostatic and bactericidal effects of EOs of *T. satureioides* leaves and flowering top on *E. coli*, *S. aureus*, *A. baumannii*, *B. cereus*, and *Enterobacter cloacae* revealed a significant antibacterial activity (inhibition zone diameters (Ф): 16 mm < Ф < 30.7 mm for leaves EOs; 22 mm < Ф < 45 mm for flowering top EOs) and a bacteriostatic effect against all tested strains except *B. cereus* [71].

Mekkaoui et al. [70] tested *in vitro* the antimicrobial effect of EOs of *T. satureioides* harvested at two different phenological stages (flowering and postflowering) against three pathogenic bacteria responsible for foodborne disease in Morocco (*E. coli*, *B. subtilis,* and *Mycobacterium smegmatis*). They showed that EOs obtained after the flowering stage were more active against the studied strains than those obtained in the flowering stage. In fact, the highest activity was shown against *M. smegmatis* followed by *B. subtilis*, while the weakest activity was noticed against *E. coli* [70]. In the same context, Oussalah et al. [72] evaluated the antibacterial potential of the *T. satureioides* flower EOs against four pathogenic bacteria including two Gram-positive bacteria: *S. aureus* and *Listeria monocytogenes* (2812 1/2a), and two Gram-negative bacteria: *E. coli* O157:H7 and *S. typhimurium* (SL 1344), using the broth microdilution method. The results indicated that *S. aureus* was the most sensitive bacterium with MIC = 0.05% (v/v) followed by *E. coli* O157:H7 and *S. typhimurium* (MIC = 0.2% (v/v)), while *L. monocytogenes* was the least sensitive bacteria to the tested EOs with MIC = 0.4% (v/v).

More interestingly, Amrouche et al. [18] investigated both *in vitro* and in a food system the antibacterial activity of *T. satureioides* EOs, extracted from the whole plant, against foodborne bacteria (*E. coli*, *S. aureus*, and *B. cereus*). The paper disc diffusion and broth microdilution methods were used for the *in vitro* test and the beef minced meat was used as food model. Thereby, the addition of *T. satureioides* EOs to inoculated beef minced meat decreased the tested strain population after 4 days of storage. Moreover, *in vitro* investigations indicated that *B. cereus* was the most sensitive bacteria (Ф = 19 mm and MIC = 1.1%), followed by *S. aureus* (Ф = 16 mm, MIC = 1.1%) and then *E. coli* (Ф = 14.25 mm, MIC = 1.25%) [18].

El Abdouni Khayari et al. [65] reported a good antibacterial activity of the *T. satureioides* aerial part EOs against *B. cereus* (Ф = 30.00 ± 0.50 mm, MIC = 2.25 mg/mL), followed by *M. luteus* (Ф = 26.70 ± 0.20 mm, MIC = 4.5 mg/mL), *L. monocytogenes* (Ф = 19.30 ± 0.60 mm, MIC = 4.5 mg/mL), *S. aureus* (Ф = 16.30 ± 2.10 mm, MIC = 4.5 mg/mL), *K. pneumoniae* (Ф = 19.00 ± 1.00 mm, MIC = 9 mg/mL), and *E. coli* (Ф = 11.70 ± 0.60 mm, MIC = 18 mg/mL), compared to standards: cefixime, gentamicin, and kanamycin, while no effect was observed against *P. aeruginosa* (ATCC 27853) [65].

Another work demonstrated an antibacterial effect of EOs extracted from wild and cultivated *T. satureioides* against Gram-positive (*S. aureus*, *M. luteus*, *B. subtilis*, and *B. cereus*) higher than against Gram-negative strains (*E. coli* (ATCC 25922), *E. coli* (CCMM B4), *Salmonella sp*., and *E. cloacae*) [15]. The highest activity was found for *M. luteus* (Ф = 49.17 ± 1.15 mm for wild TS, Ф = 29.67 ± 1.15 mm for cultivated TS), while the weakest activity was noticed against *S. aureus* (Ф = 30.33 ± 0.58 mm for wild TS, Ф = 47.83 ± 1.32 mm for cultivated TS). Moreover, a considerable activity was also noted against Gram-negative bacteria with inhibition zone diameters ranging from 19.00 ± 0.10 to 23.00 ± 1.00 mm. The microdilution approach revealed a promising antibacterial effect of the tested EOs with MIC values ranging from 0.45 to 1.78 *μ*g/mL [15]. This remarkable antibacterial effect of the *T. satureioides* EOs is concordant with the results of an earlier investigation, which indicated that *T. satureioides* E.Os inhibit the growth of a panel of microorganisms (65 Gram-positive and Gram-negative bacterial strains), among which *Aeromonas hydrophila*, *Vibrio cholera*, and *Stenotrophomonas maltophilia* were the most sensitive bacteria with respective MIC values of 0.14 ± 0.4% (v/v), 0.14 ± 0.0% (v/v), and 0.16 ± 0.0% (v/v) [74].

Recently, Meziani et al. [63] studied the antimicrobial effect of EOs and aqueous and methanolic extract from thyme leaves against *Microbacterium testaceum* and *Serratia marcescens* endophytic to date palm by using different approaches including quantitative and qualitative methods. They demonstrated that the EOs were more active against both reported bacteria than aqueous and methanolic extracts (Фs < 15 mm). Thus, these EOs have a great inhibition power against *M. testaceum* (Ф > 20 mm, MIC = 0.025%, MBC = 0.033%) and a good effect against *S. marcescens* (15 mm < Ф < 19 mm, MIC = 0.033%, MBC = 0.05%) [63].

#### 3.4.2. Antioxidant Activity

The use of *T. satureioides* as a food preservative and against several pathologic disorders in Moroccan folk medicine encouraged the research teams to study the antioxidant potential of this plant species. In fact, several works reported the antioxidant activity of *T. satureioides* EOs as well as its extracts obtained from different plant parts (aerial parts, flowering top, and leaves) using different methods such as 2,2′-azino-bis 3-ethylbenzothiazoline-6-sulphonic acid (ABTS) radical scavenging, ferric reducing power, 2,2-diphenyl-1-picrylhydrazyl (DPPH) radical scavenging, thiobarbituric acid reactive substances (TBARS), 2,2-azobis 2-amidinopropane dihydrochloride (APPH), and *β*-carotene/linoleic acid bleaching assays [13, 21, 39, 75]. All published works that studied the antioxidant activity of *T. satureioides* EOs and extracts have been listed and summarized in Table 4.

Sbayou et al. [43] reported an important antioxidant activity of the EOs of *T. satureioides* aerial parts using different methods, namely, DPPH free radical scavenging, TBARS, and *β*-carotene/linoleic acid assays. In fact, the tested EOs exhibit a strong reduction of DPPH radical (IC_50_ = 0.25 ± 0.03 mg/mL) compared with ascorbic acid (IC_50_ = 0.25 ± 0.03 mg/mL) as standard antioxidant. However, the *β*-carotene/linoleic acid assay showed a moderate antioxidant capacity (I% = 81.78 ± 0.37%) compared to BHT (I% = 98.13 ± 0.94%) as positive control. Taoufik et al. [44] also investigated the antiradical capacity of the *T. satureioides* aerial part EOs using the DPPH scavenging test and reported an interesting antioxidant effect in a concentration-dependent manner. The IC_50DPPH_ (IC_50_ = 0.81 mg/mL) value was slightly lower than the antioxidant standards BHT (IC_50_ = 0.11 mg/mL) and Covi-oxT (IC_50_ = 0.11 mg/mL).

Another study assessed the antioxidant activity of *T. satureioides* EOs using several *in vitro* assays, including ABTS, DPPH, *β*-carotene/linoleic acid bleaching, and reducing power assays, with quercetin and BHT as antioxidant standards [49]. The antiradical activity indicated that *T. satureioides* EOs exhibit stronger activity against free radical ABTS (IC_50_ = 0.15 ± 0.36 *μ*g/mL) and DPPH (IC_50_ = 0.21 ± 1.17 *μ*g/mL) than the used antioxidant standards. Moreover, *β*-carotene test and reducing power assays also showed high antioxidant activities with respective IC_50_ values of 0.21 ± 1.74 *μ*g/mL and 0.23 ± 0.67 *μ*g/mL [49]. In contrast, an investigation carried out by Alaoui-Jamali et al. [46] indicated that the *T. satureioides* aerial part EOs exhibit a moderate scavenging activity of DPPH radical (IC_50_ = 122.53 ± 2.38 *μ*g/mL). The ferric (Fe^3+^) reducing capacity assay showed similar results with an EC_50_ value equal to 177.13 ± 2.1 *μ*g/mL.

In addition, the antioxidant capacity of *T. satureioides* extracts was also studied by many researchers. Khouya and his coworkers [39] tested the antioxidant effect of ethyl acetate, methanolic, aqueous, dichloromethane, and crude extracts of *T. satureioides* aerial parts using DPPH radical scavenging, FRAP, and APPH assays and showed a higher reductive potential of these extracts than the reference compounds (Trolox). In fact, the highest reducing power of ferric metal was shown by the ethyl acetate fraction (IC_50_ = 82.69 ± 2.29 mmol Trolox/g of dry extract), and the lowest was observed for the aqueous fraction (IC_50_ = 25.46 ± 2.71 mmol Trolox/g of dry extract). The radical scavenging activity indicated that ethyl acetate fraction exerted the highest antioxidant activity with an IC_50_ value of 0.33 ± 0.02 mg/mL, followed by crude extract (IC_50_ = 0.44 ± 0.06 mg/mL), dichloromethane fraction (IC_50_ = 0.48 ± 0.05 mg/mL), and then methanolic fraction (IC_50_ = 0.71 ± 0.09 mg/mL). However, the aqueous fraction showed the weakest antiradical capacity (IC_50_ = 0.85 ± 0.06 mg/mL) [39]. Furthermore, the APPH test indicated that the addition of the tested extracts to suspensions containing erythrocyte and 2,2′-azobis 2-amidinopropane dihydrochloride (APPH) induced an increase in the hemolysis half times [39].

The antioxidant activities of *T. satureioides* extracts obtained from the aerial part were also examined by Labiad et al. [22] who reported remarkable antioxidant activities for hexane, dichloromethane, ethyl acetate, and hydro-ethanolic extracts, using ABTS radical scavenging, DPPH, and ferric reducing antioxidant power (FRAP) methods, with ascorbic acid as positive control. The hydro-ethanolic extracts exhibited the highest antiradical effect against DPPH and ABTS radicals with IC_50_ values of 3.86 ± 0.07 *μ*g/mL and 51.27 ± 0.82 *μ*g/mL, respectively, followed by dichloromethane (IC_50DPPH_ = 23.75 ± 0.67 *μ*g/mL, IC_50ABTS_ = 80.09 ± 0.65 *μ*g/mL), ethyl acetate (IC_50DPPH_ = 23.75 ± 0.67 *μ*g/mL, IC_50ABTS_ = 85.16 ± 3.22 *μ*g/mL), and then hexane extracts (IC_50DPPH_ = 275.71 ± 11.26 *μ*g/mL, IC_50ABTS_ = 127.38 ± 3.83 *μ*g/mL). Moreover, the hydro-ethanolic extract also exerted a great FRAP activity (233.292 ± 0.377 mg equivalent Ascorbic acid/g of extract). However, the hexane extract showed the lowest FRAP capacity (97.819 ± 0.377 mg equivalent ascorbic acid/g of extract) [22].

In a recent study, Hmidani et al. [76] measured the capacity of aqueous extract of *T. satureioides* to scavenge the generated radical ABTS+•, using ABTS assay, and showed significant scavenging activity of this extract (IC_50ABTS_ = 14.65 ± 0.36 *μ*g/ml) compared to ascorbic acid as standard (IC_50_ = 1.96 ± 0.1 *μ*g/ml). These findings support those obtained by Khouya et al. [24], which showed a considerable antioxidant activity of the *T. satureioides* aerial part aqueous extract. Indeed, the tested aqueous extracts displayed potent scavenging activity against DPPH radical with an IC_50_ value equal to 0.44 ± 0.01 mg/mL TAE and a higher reducing power of ferric complex (40.14 ± 4.55 mmol Trolox/gTAE) than the Trolox used as positive control (44.33 ± 7.55 mmol Trolox/gTAE). Moreover, the aqueous extracts of *T. satureioides* exerted a potent protective potential against hemolysis of erythrocytes according to APPH test results [24]. These considerable antioxidant effects of the aqueous extracts were attributed to their high phenolic content [24].

#### 3.4.3. Antifungal Activity

The antifungal activity of *T. satureioides*, especially its essential oils against several pathogenic fungal, has been reported in the literature [19, 50, 78, 79].

Boukhira et al. [20] evaluated the antifungal activity of *T. satureioides* EOs obtained from aerial parts against a yeast, *Candida albicans* (ATCC-10231), and a mould *Aspergillus brasiliensis* (ATCC-16404), using radial growth inhibition and broth microdilution assays. This study showed that the studied EOs exert effective effect against *C. albicans* (Ф = 24.67 ± 0.67 mm, MIC = 0.6 *μ*l/mL) and *A. brasiliensis* (MIC = 1.3 *μ*l/mL).

Asdadi et al. [19] studied the anticandidal activity of *T. satureioides* EOs (10 *μ*l) against nosocomial fluconazole-resistant strains (*Candida dubliniensis, C. albicans*, *C. glabrata*, and *C. krusei*) using disc diffusion and microdilution methods, with fluconazole (10 *μ*l) and amphotericin B (10 *μ*l) as positive controls. According to this study, *C. dubliniensis* was the most sensitive strain to the tested EOs (Ф = 85 mm), followed by *C. krusei* (Ф = 67 mm), while *C. albicans* and *C. glabrata* were the least sensitive fungal strains with Ф of 53 mm and 49 mm, respectively. The microdilution assays showed an interesting anticandidal effect with minimal fungicidal concentration (MFC) values ranging between 0.3300 mg/mL and 0.9062 mg/ml [19].

In addition, El Bouzidi et al. [15] assessed the anticandidal activity of *T. satureioides* EOs obtained from aerial parts against four candida species, including *C. albicans*, *C. glabrata, C. parapsilosis*, and *C. krusei* using disc diffusion and microdilution assays, and fluconazol (40 *μ*l) as reference. The findings of this study showed that all tested strains were more sensitive to the tested EOs (37.67 ± 1.53 mm < Ф < 42.00 ± 1.00 mm) than to the synthetic fungicide (fluconazol) used as a positive control (26.50 ± 0.50 mm < Ф < 29.83 ± 1.15 mm).

More interestingly, Salhi et al. [50] reported the antifungal activity of four chemotypes of *T. satureioides* EOs from aerial parts, namely, borneol/*α*-terpineol, borneol/carvacrol/*α*-terpineol, borneol/carvacrol/thymol, and borneol/camphene/*α*-terpineol against fungal strains responsible of wood damages (*Coniophora puteana* BAM Ebw. 15, *Gloeophyllum trabeum* BAM Ebw.109, *Oligoporus placenta* FPRL. 280, and *Trametes versicolor* CTB 863). The studied samples were harvested from four different locations in Southwest Morocco (Oulad Berhil, Amskroud-East, Aoulouz, and Timoulay Aksri), and qualitative and quantitative assays were used for antifungal screening [50]. The results showed that, at a concentration of 1/500 (v/v), all tested chemotypes inhibit the growth of the tested wood-decaying fungi. However, the investigated chemotypes exhibited a variable degree of the antifungal effect. Therefore, the chemotype borneol/carvacrol/thymol was the most active against the tested strains, followed by borneol/camphene/*α*-terpineol, borneol/*α*-terpineol, and borneol/carvacrol/*α*-terpineol. Moreover, the highest antifungal activity was noticed against *G. trabeum* with MIC ranging between 1/1500 v/v and 1/500 (v/v), followed by *C. puteana* (1/1250 (v/v) <MIC< 1/500 (v/v)), *T. versicolor* (MIC = 1/500 (v/v)), and *O. placenta* (MIC = 1/500 (v/v)) [50].

In the same context, Rahmouni et al. [42] reported fungicide effect of *T. satureioides* EOs and their major components (thymol, *α*-terpineol, carvacrol, and borneol) against a phytopathogenic fungus responsible for fusarium wilt on date palm in Morocco, named *Fusarium oxysporum f. sp. Albedinis*. The results of this study showed that these EOs as well as their major compounds inhibited noticeably the mycelia growth of *Fusarium oxysporum f. sp. Albedinis* in a concentration-dependent manner. The maximal fungicidal effect of the studied compounds was noticed by thymol with a minimum fungicidal concentration (MFC) value of 03.08 *μ*l/mL, followed by *α*-terpineol (MFC = 12.20 *μ*l/mL), carvacrol (MFC = 16.96 *μ*l/mL), and borneol (MFC = 22.73 *μ*l/mL) [42]. Furthermore, *T. satureioides* EO was found to inhibit spore germination of phytopathogenic fungi of citrus, namely, *Penicillium digitatum*, *P. italicum*, and *Galactomyces citriaurantii* at concentrations greater than 500 *μ*l/mL [79].

Recently, El-Bakkal et al. [21] tested the antifungal effect of the EOs of *T. satureioides* aerial parts against *Botrytis cinerea*, *P. digitatum*, and *Verticillium dahliae* using disc diffusion method and fluconazol (40 *μ*g/disc) as standard antifungal drug. Their results showed a promising antifungal effect, of the studied EOs, against the three tested strains, with inhibition zone diameters ranging from 31.50 ± 1.32 mm to 36.27 ± 1.15 mm compared to fluconazol (25.50 ± 0.50 < Ф < 28.00 ± 0.50).

#### 3.4.4. Anti-Inflammatory Activity

Inflammation is a complex biological process that maintains homeostasis of the organism in response to multiple injuries such as infection, trauma, or immune reaction. It is characterized by pain, heat, redness, and swelling [80].

Inflammation is related to the occurrence of several human pathologies, including heart diseases, Alzheimer's disease, and cancer [81–83]. The mechanisms of the anti-inflammatory response involve various mediators such as phospholipase A2 activation, cytokines, chemokines, reactive oxygen species (ROS) generation, macrophages and mast cells, platelet-activating factor, and nitric oxide (NO) [83].

Khouya et al. [39] have evaluated *in vivo* the anti-inflammatory activity of *T. satureioides* crude extracts and fractions (dichloromethane, ethyl acetate, methanol, and aqueous) using croton-oil-induced ear oedema and carrageenan-induced paw oedema in mice and rats. The results of this study showed that topical applications of the dichloromethane and ethyl acetate fractions (1 mg/ear) reduced significantly ear oedema volume of 31.60% and 27.16%, respectively, after 4 h of treatment. The crude extracts exhibited the greatest activity, and its tropical application decreased significantly ear oedema (29.67%) 8 h after treatment. However, the methanol and aqueous fractions did not decrease ear oedema. Moreover, the results of carrageenan oedema assay showed that the ethyl acetate and methanol fractions (60 mg/kg) reduced significantly oedema induced by carrageenan during the first phase (16.40 ± 0.33% and 14.51 ± 1.40%, respectively) [39]. This study confirmed results obtained by the same authors, indicating that aqueous extracts of *T. satureioides* exhibited a remarkable anti-inflammatory effect in carrageenan-induced rats paw edema and in croton oil-induced mice ear edema [24]. In another previous study, Ismaili et al. [14] investigated the *in vivo* topical anti-inflammatory effect of methanol and chloroform extracts of *T. satureioides* leaves, using the croton oil ear test in mice, and showed that chloroform extract induced significant edema inhibition (at a inhibition dose ID_50_ of 282 *μ*g·cm^−2^), only three times lower than that of the standard conventional drug indomethacin used as positive control (ID50 = 93 *μ*g·cm^−2^), while the methanolic extract did not show any topical anti-inflammatory activity.

#### 3.4.5. Antiparasitic Effect

*T. satureioides* EOs from different plant parts were studied against a number of human, virus, and plant parasites. Indeed, Pavela [84] assessed the toxicity of *T. satureioides* EOs against the larvae of *Culex quinquefasciatus* Say (Diptera: Culicidae) and showed its effective larvicidal property with respective lethal concentrations (IC_50_ and IC_90_) of 44 *μ*g/ml and 81.5 *μ*g/ml.

Kasrati et al. [49] reported a considerable insecticidal activity of *T. satureioides* EOs against adults of pest *Tribolium castaneum* responsible for stored-product deterioration (lethal dose values of LD_50_ = 0.315 *μ*l/cm^2^ and LD_90_ = 0.71 *μ*l/cm^2^). Moreover, Santana et al. [25] examined the toxicity of *T. satureioides* EOs against insect pest's larvae of *Spodoptera littoralis*, insect adults of *Myzus persicae* and *Rhopalosiphum padi*, as well as against adults and eggs of root-knot nematodes *Meloidogyne javanica*. A strong antifeedant effect of *T. satureioides* EOs was observed against *S. littoralis* larvae (EC_50_ = 36.9 ± 22.7 *μ*g/cm2), *M. persicae* adults (EC_50_ = 53.53 ± 6.5 *μ*g/cm^2^), and *R. padi* (EC_50_ = 49.0 ± 6.6 *μ*g/cm^2^). Additionally, an important nematicidal effect was noticed against the tested *M. javanica* at two different development stages: second-stage juveniles (J2) (LC_50_ = 0.1 mg/mL and LC_90_ = 0.2 mg/mL) and eggs (mortality rate of 38.9% after 7 days) [25].

Another study conducted by Avato et al. [26] showed that *T. satureioides* EOs exhibit a promising nematicidal activity against *Meloidogyne incognita* juveniles (mortality rate of 10.6 ± 0.7%) and adults of *Pratylenchus vulnus* (100 ± 0.0%) and *Xiphinema index* (14.9 ± 0.7%) and that, after 48 h, this effect was dose-dependent.

The acaricidal activity of *T. satureioides* EOs was also reported in the literature. Ramzi et al. [85] studied the effect of the EOs of *T. satureioides* aerial parts against adults of *Varroa destructor* (Acari: Varroidae) and indicated an interesting mortality rate of 50% after 24 h and 80% after 48 h. Additionally, *T. satureioides* EO was shown to destroy completely the wheat pest *Sitophilus oryzae* (coleopters) at a concentration of 2.4 × 10^−2^ *μ*l/cm^3^ after 24 h [86].

#### 3.4.6. Other Pharmacological Properties

*T. satureioides* was also reported to exhibit other pharmacological properties such as anticancer, antidiabetic, and hypolipidemic effects.

Jaafari et al. [23] evaluated the *in vitro* antitumor activity of *T. satureioides* EOs collected in different regions (High Atlas of Morocco, Bin Elwidane-Beni Mellal, and Tiznit) on P815 mastocytoma cell line using the 3-(4,5-di-methylthiazol-2-yl)-2,5-diphenyl tetrazolium bromide (MTT) assay. The results showed that all tested EOs exhibit an important cytotoxic effect against P815 cell line with IC_50_ values from 0.225% (v/v) to 0.24% (v/v). The antiproliferative effect of *T. satureioides* crude extracts was studied against MCF-7 breast cancer cell line using MTT assay and showed their strong inhibition with a half-inhibitory concentration (IC_50_) value of 37.5 ± 4.02 *μ*g/mL [24].

Kabbaoui et al. [2] investigated the antidiabetic effect of *T. satureioides* aqueous extracts obtained from the aerial parts on streptozotocin- (STZ-) induced diabetic rats via the administration of an oral concentration of 500 mg/kg. As a result, *T. satureioides* aqueous extracts decreased significantly blood glucose levels and improved body weight and glucose tolerance in STZ-diabetic rats.

## 4. Conclusion and Perspectives

This scientific review reports the ethnomedicinal uses, chemical profile, and pharmacological properties of an endemic Moroccan medicinal plant: *T. satureioides*. This plant is widely used in Moroccan traditional medicine to treat several diseases such as hypertension, diabetes, skin ailments, and bronchitis.

Indeed, several investigations have demonstrated that *T. satureioides* exhibits numerous biological activities, including antibacterial, antifungal, antioxidant, anti-inflammatory, anticancer, antidiabetic, and antiparasitic activities. These pharmacological effects have proven the traditional uses of *T. satureioides*. However, the evidence supporting the traditional practices such as skin disorders, hypertension, influenza, and visual ailments of modern pharmacology is still limited. In this regard, we invite research groups to conduct further studies on the antiviral, antileishmanial, and hypotensive effects of *T. satureioides*. Furthermore, the pharmacological mechanisms of action, of this plant, on molecular targets need to be explored using current experimental assessments such as network pharmacology, proteomic, and pharmacokinetic. Additionally, an appropriate pharmacological approach should be considered for providing comprehensive pharmacological information for *T. satureioides*. Moreover, *T. satureioides* have shown interesting biological effects against some related oxidative stress such as inflammation and cancer. Accordingly, extensive clinical studies should be carried out to determine pharmacodynamic and pharmacokinetic parameters in order to develop drug from *T. satureioides*.

The phytochemical analysis using different chromatographic tools such as GC-MS and HPLC revealed the presence of a plethora of bioactive compounds mainly belonging to the terpenoids class in the essential oils of *T. satureioides*. This chemical diversity varied depending on plant's part used, season's harvest, plant's origin, as well as extraction and storage conditions. However, although numerous bioactive compounds have been isolated and identified from *T. satureioides* essential oils, few pure components have been assessed for their pharmacological effects. Furthermore, few studies have investigated the phenolic content of *T. satureioides* extracts. Therefore, further efforts should be focused on such area in order to determine in detail the phenolic profile of this species using different extraction solvents and the current spectroscopic tools such as HPLC-DAD, infrared (IR), and ^1^H NMR technique.

Finally, the acute, subacute, and subchronic toxicity tests are strongly required to verify the innocuity and the safety of this plant.

## Figures and Tables

**Table 1:** Ethnomedicinal use of *T. satureioides*.

Study area	Parts used	Preparation method	Medicinal use	References
Agadir-Ida-Ou Tanane (Morocco)	Aerial parts	Infusion, decoction, cataplasms, and fumigation	Gastrointestinal complaints, influenza, colds, fever, headaches, affections of the annex glands of the digestive tract, respiratory problems, and menstruation pain in women	[5]

Agadir-Ida-Ou-Tanane Province (Southwest Morocco)	Whole plant, flowers, leaves, and stems	Infusion	Respiratory, digestive, skin, circulatory, genital, nervous, visual, and urinary problems	[12]

Beni Mellal (Morocco)	Leaves	Decoction and infusion	Diabetes	[28]

High Atlas mountains (Morocco)	Whole plant	Powder	Gastrointestinal ailments (stomach ache and intestinal trouble) and respiratory disorders such as colds and coughs	[29]

Haouz-Rhamna region (Morocco)	Leaves	Decoction and infusion	Diabetes	[9]

Er-Rich region (High Atlas of Morocco)	Aerial parts	Decoction and infusion	Gastric disorders, chills, cold, fever, headaches, digestive infections, and pain, and it is also used as an antispasmodic agent	[11]

Er-Rich region	Aerial parts	Fumigation	Respiratory diseases, digestive ailments	[11]

Agadir region (Morocco)	Leaves	Infusion	Diabetes	[30]

Chtouka Ait Baha and Tiznit (Morocco)	Leaves	Infusion, maceration, and powder	Diabetes	[31]

Western Middle Atlas region (Morocco)	Leaves and stems	Infusion	Gastrointestinal disorders (bloating, diarrhea)	[32]

Zagora (Morocco)	Leaves	Decoction and powder	Diabetes and used as antinociceptive agent	[33]

Azilal (Morocco)	Aerial parts	Fumigation, infusion	Digestive ailment, colds, and coughs	[34]

Seksaoua region, Western High Atlas (Morocco)	Leaves	Decoction	Cooling, pharyngitis, cough, and influenza	[10]

Morocco	Leaves, aerial part	Decoction, infusion	Coughs and bronchitis	[1]

Beni Mellal region (Morocco)	Whole plant	Infusion	Gastrointestinal ailments	[8]

Berber Peoples of Morocco	Aerial parts	Infusion	Treatment of cold and colic and as food disinfectant	[35]

Marrakech (Morocco)	Aerial parts	Decoction	Digestive ailments	[36]

Errachidia Province (Morocco)	Leaves, flower	Decoction	Arterial hypertension	[37]

Tata Province, Morocco	Aerial part	Decoction	Hypotensive, digestive ailments, diabetes, colds	[6]

Region of Middle Oum Rbia (Morocco)	Whole plant, leaves	Not reported	Dermatological, immune, and digestive and respiratory ailments	[7]

**Table 2:** Chemical compounds from *T. satureioides*.

No.	Compounds	Parts used	Extracts	References
**1**	Apigenin	Leaves	Alcohol	[38]
**2**	Luteolin	Leaves, Aerial parts	Alcohol, methanol	[14, 38]
**3**	Eriodictyol	Leaves	Methanol	[14]
**4**	Thymonin	Leaves	Methanol	[14]
**5**	Quercetin	Aerial parts	Crude extracts, ethyl acetate, methanol	[39]
**6**	Hesperetin	Aerial parts	Crude extracts, ethyl acetate, methanol, aqueous	[13, 27, 39]
**7**	Luteolin-3′-O-glucuronide	Leaves	Methanol	[14]
**8**	Apigenin-7-O-glucoside	Aerial parts	Dichloromethane	[39]
**9**	Hyperoside	Aerial parts	Dichloromethane, ethyl acetate, methanol	[39]
**10**	Luteolin-7-O-glucoside	Leaves, Aerial parts	Aqueous, methanol	[13, 14, 24]
**11**	Eriodictyol-7-O-glucoside	Leaves	Methanol	[14]
**12**	Caffeic acid	Aerial parts, Leaves	Alcohol, ethyl acetate, methanol	[38, 39]
**13**	*p*-Coumaric acid	Leaves	Alcohol	[38]
**14**	Ferulic acid	Leaves	Alcohol	[38]
**15**	Rosmarinic acid	Aerial parts	Crude extracts	[27, 39]
**16**	Chlorogenic acid	Leaves	Alcohol	[38]
**17**	Ursolic acid	Leaves	Chloroform	[14]
**18**	Oleanolic acids	Leaves	Chloroform	[14]
**19**	(*E*)-Linalool oxide	Aerial parts	EOs	[40]
**20**	(*E*)-p-Menthan-2-one	Aerial parts	EOs	[40]
**21**	(*E*)-Sabinene hydrate	Aerial parts	EOs	[40]
**22**	(*E*)-Verbenol	Aerial parts	EOs	[40]
**23**	(*E*)-*β*-Ocimene	Flowering top	EOs	[41]
**24**	(*Z*)-Dihydrocarvone	Aerial parts	EOs	[40]
**25**	(*Z*)-Sabinene hydrate	Aerial parts	EOs	[40]
**26**	1,10-di-epi-Cubenol	Whole plant, aerial parts	EOs	[19, 41]
**27**	1,8 Cineole	Aerial parts	EOs	[40]
**28**	Thymol methyl ether (2-Isopropyl-5-methylanisole)	Aerial parts	EOs	[40]
**29**	3-Octanol	Whole plant, aerial parts, flowering top	EOs, petroleum ether, ethyl acetate	[19, 23, 41]
**30**	3-Tetradecen-5-yne	Leaves	EOs	[42]
**31**	3-Thujen-2-one	Aerial parts	EOs	[16]
**32**	3-*δ*-Carene	Aerial parts	EOs	[40]
**33**	Alloaromadendrene	Aerial parts, flowering top	EOs	[40, 41]
**34**	Alloocimene	Aerial parts	Petroleum ether, EOs	[23, 43]
**35**	Aromadendrene	Aerial parts	EOs	[40]
**36**	Bicyclogermacrene	Flowering top, aerial parts	EOs	[41, 44]
**37**	Borneol	Aerial parts, flowering top	EOs, petroleum ether, ethyl acetate	[23, 41, 45]
**38**	Bornyl acetate	Aerial parts, flowering top	EOs, petroleum ether, ethyl acetate	[23, 41, 45]
**39**	Bornyl formate	Aerial parts	EOs	[16]
**40**	Calamenene	Aerial parts	EOs	[40]
**41**	Calarene	Aerial parts	EOs, petroleum ether, ethyl acetate	[23, 43]
**42**	Camphene	Aerial parts, whole plant, flowering top	EOs, petroleum ether, ethyl acetate	[19, 41, 46]
**43**	Camphene hydrate	Aerial parts	EOs	[44]
**44**	Camphenilone	Aerial parts	EOs	[44]
**45**	Camphor	Aerial parts, flowering top	EOs	[41, 47]
**46**	Carvacrol (5-isopropyl-2-methylphenol)	Aerial parts, flowering top	EOs, petroleum ether, ethyl acetate	[23, 41, 47]
**47**	Carvacrol methyl ether	Aerial parts	EOs, petroleum ether, ethyl acetate	[15, 21, 23]
**48**	Carvenone	Aerial parts	EOs, petroleum ether, ethyl acetate	[23, 44]
**49**	Carveol	Aerial parts	EOs	[40]
**50**	Carvone	Aerial parts	EOs	[40]
**51**	Caryophyllene oxide	Aerial parts	EOs, petroleum ether, ethyl acetate	[23, 40]
**52**	Cedrene oxide	Aerial parts	EOs	[48]
**53**	*cis*-Linalool oxide	Aerial parts, flowering top	EOs	[43, 44]
**54**	*cis*-Ocimene	Aerial parts	EOs	[44]
**55**	*cis*-*α*-Bisabolene	Leaves	EOs	[42]
**56**	Copaene	Aerial parts	EOs	[40]
**57**	Crithmene	Aerial parts	EOs	[47]
**58**	Dehydro-p-cymene	Aerial parts	EOs	[40]
**59**	Dihydrocarvone 1	Aerial parts	EOs	[40]
**60**	Dihydrocarvone 2	Aerial parts	EOs	[40]
**61**	Dodecamethylcyclohexasiloxane	Aerial parts	EOs, petroleum ether	[23, 43]
**62**	Eucalyptol	Leaves	EOs	[42]
**63**	Eugenol	Aerial parts, whole plant	EOs	[19, 44]
**64**	Fenchone	Flowering top	EOs	[16]
**65**	Geraniol formate	Aerial parts	Ethyl acetate	[23]
**66**	Geranyl linalool	Aerial parts	EOs	[40]
**67**	Germacrene-D-4-ol	Flowering top	EOs	[41]
**68**	Germacrene	Aerial parts	EOs	[15]
**69**	Guaia-3,9-diene	Aerial parts	Petroleum ether, EOs	[23, 43, 48]
**70**	Guaiazulene	Aerial parts	EOs	[40]
**71**	Hexahydroindan	Aerial parts	EOs, petroleum ether	[23, 43]
**72**	Hotrienol	Leaves	EOs	[42]
**73**	Isoaromadendrene epoxide	Aerial parts	Petroleum ether, EOs	[23, 40]
**74**	Isoborneol	Aerial parts	EOs	[40]
**75**	Isobornyl acetate	Aerial parts	EOs, petroleum ether	[23, 43]
**76**	Isobornyl formate	Aerial parts	EOs, petroleum ether, ethyl acetate	[23, 44]
**77**	Isoledene	Leaves	EOs	[42]
**78**	Isothymol methyl ether	Leaves	EOs	[42]
**79**	Ledene	Aerial parts	EOs, petroleum ether	[23, 43]
**80**	Ledol 6-epi-cubenol	Flowering top	EOs	[41]
**81**	Limonene	Aerial parts, flowering top	EOs	[15, 41]
**82**	Linalool	Aerial parts, flowering top	EOs	[41, 45]
**83**	Linalyl propionate	Aerial parts	Ethyl acetate, petroleum ether	[23]
**84**	Thymol methyl ether	Aerial parts, flowering top	EOs	[40, 41]
**85**	Myrcene	Aerial parts, flowering top	EOs	[15, 41]
**86**	Octan-3-one	Aerial parts	EOs	[45]
**87**	Octen-3-ol	Aerial parts	EOs	[40]
**88**	*p*-Cymen-8-ol (2-(4-methylphenyl) propan-2-ol)	Flowering top	EOs	[41]
**89**	p-Cymene	Aerial parts, flowering top	EOs, petroleum ether, ethyl acetate	[23, 41, 45]
**90**	Pentasiloxane	Aerial parts	Petroleum ether	[23]
**91**	Pinocarveol	Aerial parts	EOs	[40]
**92**	*p*-Menth-2-en-1-ol	Aerial parts	EOs	[40]
	*p*-Mentha-1.8-diene	Aerial parts	Petroleum ether, ethyl acetate	[23]
**93**	Sabinene	Aerial parts	EOs	[49]
**94**	Santolina triene	Aerial parts	EO, petroleum ether	[23, 43]
**95**	Spathulenol	Aerial parts	EOs	[40]
**96**	*tau*-Cadinol	Aerial parts, whole plant	EOs	[19, 44]
**97**	Terpinen-4-ol	Aerial parts, whole plant	EOs	[19, 44]
**98**	Terpinolene	Aerial parts	EOs	[40]
**99**	Thuja-2,4(10)-diene	Aerial parts	EOs	[40]
**100**	Thujone	Aerial parts, whole plant	EOs	[19, 44]
**101**	Thymol	Aerial parts, flowering top	EOs, petroleum ether, ethyl acetate	[23, 41, 42]
**102**	Trans-1,2-diphenylcyclobutane	Aerial parts	Petroleum ether	[23]
**103**	trans-Pinocarveol	Flowering top	EOs	[41]
**104**	trans-Sabinene hydrate	Flowering top	EOs	[41]
**105**	Tricyclene	Aerial parts, whole plant, flowering top	EOs	[19, 41, 46]
**106**	Valencene	Aerial parts	EOs, petroleum ether	[23, 43]
**107**	*α*-Amorphene	Leaves	EOs	[42]
**108**	*α*-Cadinol	Aerial parts	EOs	[21]
**109**	*α*-Campholenal	Aerial parts	EOs	[44]
**110**	*α*-Campholene aldehyde	Aerial parts	EOs, petroleum ether	[23, 43]
**111**	*α*-Cubebene	Aerial parts	EOs	[40]
**112**	*α*-Curcumene	Aerial parts	EOs	[44]
**113**	*α*-Ferulene	Aerial parts	EOs, petroleum ether	[23, 43]
**114**	*α*-Guajene	Aerial parts	EOs, petroleum ether	[23, 43]
**115**	*α*-Gurjunene	Aerial parts	EOs	[44]
**116**	*α*-Humulene	Aerial parts, flowering top	EOs	[15, 41]
	*α*-Muurolene	Aerial parts	Petroleum ether	[23]
**117**	*α*-Panasisen	Aerial parts	EOs	[40]
**118**	*α*-Pentasiloxane	Aerial parts	EOs	[43]
**119**	*α*-Phellandrene	Aerial parts	EOs, petroleum ether	[23, 43]
**120**	*α*-Pinene	Aerial parts, whole plant	EOs	[19, 46]
**121**	*α*-Terpineol	Aerial parts	EOs	[50]
**122**	*α*-Thujene	Aerial parts, whole plant, flowering top	EOs	[19, 41, 46]
**123**	*β*-Bourbonene	Flowering top	EOs	[41]
**124**	*β*-Caryophyllene	Aerial parts	EOs	[47]
**125**	*β*-Cubebene	Aerial parts	EOs	[40]
**126**	*β*-Gurjunene	Aerial parts	EOs	[40]
**127**	*β*-Ionone	Aerial parts	EOs	[40]
**128**	*β*-Linalool	Aerial parts	Ethyl acetate	[23]
**129**	*β*-Oplopenone	Aerial parts, whole plant	EOs	[19, 44]
**130**	*β*-Patchoulene	Aerial parts	EOs	[40]
**131**	*β*-Phellandrene	Flowering top, aerial parts	EOs	[41, 48]
**132**	*β*-Pinene	Aerial parts, flowering top	EOs, petroleum ether, ethyl acetate	[16, 23, 41]
**133**	*γ*-Cadinene	Aerial parts, flowering top	EOs	[41, 47]
**134**	*γ*-Costol	Aerial parts	EOs	[48]
**135**	*γ*-Methylionone	Aerial parts	EOs	[48]
**136**	*γ*-Muurolene	Aerial parts, flowering top	EOs	[41, 42]
**137**	*τ-*Muurolol	Aerial parts	Petroleum ether	[23]
**138**	*γ*-Terpinene	Aerial parts	EOs, petroleum ether, ethyl acetate	[15, 23, 50]
**139**	*δ*-Cadinene	Aerial parts, flowering top	EOs	[41, 50]

**Table 3:** Antibacterial activity of *T. satureioides*.

Parts used	Extracts	Methods used	Bacteria tested	Key results	References
Aerial parts	Essential oil (0.93%)	Broth microdilution method	*Staphylococcus aureus* CCMM B_3_	MIC = 4.50 mg/mL	[65]
			*Micrococcus luteus* ATCC 10240	MIC = 4.50 mg/mL	
			*Bacillus cereus* ATCC 14579	MIC = 2.50 mg/mL	
			*Listeria monocytogenes* ATCC 19115	MIC = 4.50 mg/mL	
			*Escherichia coli* ATCC 25922	MIC = 18.00 mg/mL	
			*Pseudomonas aeruginosa* ATCC 27853	MIC > 18.4 mg/mL	
			*Klebsiella pneumoniae*	MIC = 9 mg/mL	

Stem	Aqueous extract	Paper disc diffusion assay	*Clavibacter michiganensis subsp. michiganensis* H195 isolate	Ф = 23.3 ± 2.4 mm	[66]

Leaves	Aqueous extract	Paper disc diffusion assay	*Clavibacter michiganensis subsp. michiganensis* H195 isolate	Ф = 16.4 ± 0.5 mm	[66]

Aerial part	Essential oil	Agar disc diffusion method	*Staphylococcus aureus* ATCC 29213	Ф = 34.67 ± 0.33 mm	[20]
			*Escherichia coli* ATCC 25922	Ф = 20.67 ± 0.27 mm	
			*Pseudomonas aeruginosa* ATCC 27853	Ф = 7.00 ± 0.00 mm	

Aerial part	Essential oil	Microdilution assay	*Escherichia coli* ATCC 25922	MIC = 0.125%	[67]
			*Pseudomonas aeruginosa* ATCC 27853	MIC = 1%	
			*Micrococcus luteus* ATCC 14452	MIC = 0.03%	
			*Staphylococcus aureus* ATCC 29213	MIC = 0.03%	
			*Bacillus subtilis* ATCC 6633	MIC = 0.03%	
			*Salmonella typhimurium*	MIC = 0.25%	
			*Bacillus cereus*	MIC = 0.015%	

Whole plant	Essential oil (1.78%)	Microdilution assay	*Staphylococcus aureus* ATCC 25923	MIC = 2.5 *μ*l/mL	[17]
			*Streptococcus fasciens* ATCC 29212)	MIC = 2.5 *μ*l/mL	
			*Escherichia coli* ATCC 4157	MIC = 5 *μ*l/mL	
			*Pseudomonas aeruginosa* ATCC 27853	MIC = 10 *μ*l/mL	

Aerial part	Essential oil	Agar diffusion method Broth microdilution method	*Escherichia coli* ATCC 25921	Ф = 15 ± 0 mm MIC = 0.625 *μ*l/mL MBC = 0.625 *μ*l/mL	[43]
			*Escherichia coli*	Ф = 21 ± 0 mm MIC = 125 *μ*l/mL MBC = 125 *μ*l/mL	
			*Pseudomonas aeruginosa* ATCC 27853	Ф = 0 ± 0 mm MIC > 20 *μ*l/mL MBC > 20 *μ*l/mL	
			*Pseudomonas aeruginosa*	Ф = 0 ± 0 mm MIC > 20 *μ*l/mL MBC > 20 *μ*l/mL	
			*Enterobacter cloacae*	Ф = 15.5 ± 0.7 mm MIC = 0.625 *μ*l/mL MBC = 0.625 *μ*l/mL	
			*Staphylococcus aureus* ATCC 29213	Ф = 23 ± 0 mm MIC = 0.312 *μ*l/mL MBC = 0.312 *μ*l/mL	
			*Staphylococcus aureus*	Ф = 15 ± 0 mm MIC = 0.625 *μ*l/mL MBC = 0.625 *μ*l/mL	
			*Enterococcus faecium*	Ф = 16 ± 0 mm MIC = 125 *μ*l/mL MBC = 125 *μ*l/mL	

Aerial part	Essential oil	Agar-dilution method	*Enterobacter cloacae* (clinical strain, nosoco.tech Abdel1)	MIC = 2.9 *μ*g/mL	[68]
			*Escherichia coli* CIP 54127	MIC = 2.9 *μ*g/mL	
			*Klebsiella pneumoniae* CIP 104216	MIC = 2.9 *μ*g/mL	
			*P. aeruginosa* ATCC 15442	MIC = 11.7 *μ*g/mL	
			*Salmonella typhimurium* ATCC 133115	MIC = 2.9 *μ*g/mL	
			*Listeria monocytogenes* ATCC 35152	MIC = 5.8 *μ*g/mL	
			Methicillin-resistant *Staphylococcus aureus* (MRSA)	MIC = 2.9 *μ*g/mL	
			*Enterococcus faecalis* CIP A185	MIC = 5.8 *μ*g/mL	
			*Streptococcus equinus* CIP 56.23	MIC = 2.9 *μ*g/mL	
			*Streptococcus pyogenes* CIP 70.3	MIC = 2.9 *μ*g/mL	

Aerial parts	Essential oil	Agar diffusion method Broth microdilution method	*Escherichia coli* ATCC25922	Ф = 12.3 ± 0.6 mm MIC = 1.5%	[40]
			Non-O1 *Vibrio cholera*	Ф = 33.3 ± 2.9 mm MIC = 0.5%	
			*Pseudomonas aeruginosa* CCMMB11	Ф = 11.7 ± 1.5 mm MIC = 1.5%	
			*Enterobacter cloacae*	Ф = 11.7 ± 0.6 mm MIC = 1.5%	
			*Klebsiella pneumoniae*	Ф = 13.3 ± 0.6 mm MIC = 1.5%	
			*Staphylococcus aureus* CCMMB3	Ф = 29.3 ± 2.1 mm MIC = 0.125%	
			*Bacillus subtilis* ATCC9524	Ф = 34.3 ± 1.1 mm MIC = 0.003%	
			*Bacillus cereus* ATCC14579	Ф = 30 ± 0 mm MIC = 0.003%	

Aerial parts	Essential oil (1.86%)	Agar disc diffusion Agar dilution technique	*Staphylococcus aureus* CCMM B_3_	Ф = 29.67 ± 1.15 mm MIC = 1.78 mg/mL	[15]
			*Bacillus subtilis* ATCC 9524	Ф = 43.67 ± 1.53 mm MIC = 0.89 mg/mL	
			*Bacillus cereus* ATCC 14579	Ф = 43.67 ± 1.53 mm MIC = 0.89 mg/mL	
			*Micrococcus luteus* ATCC 10240	Ф = 42.00 ± 1.73 mm MIC = 0.45 mg/mL	
			*Escherichia coli* ATCC 25922	Ф = 22.5 ± 1.32 mm MIC = 1.78 mg/mL	
			*Escherichia coli* CCMM B_4_	Ф = 23.00 ± 1.00 mm MIC = 1.78 mg/mL	
			*Salmonella sp.* CCMM B17	Ф = 22.33 ± 0.58 mm MIC = 1.78 mg/mL	
			*Enterobacter cloacae*	Ф = 21.00 ± 1.00 mm MIC = 1.78 mg/mL	

Leaves	Essential oil (2.95%(v/w))	Agar diffusion assay	*Bacillus cereus*	Ф = 12.5 mm MIC = 80 *μ*g/mL	[41]
			*Staphylococcus aureus* ATCC 5638	Ф = 8.0 mm MIC = 640 *μ*g/mL	
			*Listeria monocytogenes*	Ф = 14.5 mm MIC = 40 *μ*g/mL	
			*Aeromonas hydrophila*	Ф = 11.8 mm MIC = 160 *μ*g/mL	
			*Escherichia coli*	Ф = 9.0 mm MIC = 320 *μ*g/mL	
			*Proteus vulgaris*	Ф = 7.4 mm MIC = 640 *μ*g/mL	
			*Pseudomonas aeruginosa*	ND MIC = 1280 *μ*g/mL	
			*Pseudomonas fluorescens*	Ф = 7.2 mm MIC = 640 *μ*g/mL	
			*Salmonella abony*	Ф = 7.8 mm MIC = 640 *μ*g/mL	

Inflorescences (flowers)	Essential oil (2.95% (v/))		*Bacillus cereus*	Ф = 13.8 mm MIC = 80 *μ*g/mL	[41]
			*Staphylococcus aureus* ATCC5638	Ф = 8.4 mm MIC = 320 *μ*g/mL	
			*Listeria monocytogenes*	Ф = 15.2 mm MIC = 40 *μ*g/mL	
			*Aeromonas hydrophila*	Ф = 14.2 mm; MIC = 80 *μ*g/mL	
			*Escherichia coli*	Ф = 10.4 mm MIC = 160 *μ*g/mL	
			*Proteus vulgaris*	Ф = 8.2 mm MIC = 320 *μ*g/mL	
			*Pseudomonas aeruginosa*	Ф = 6.8 mm MIC = 640 *μ*g/mL	
			*Pseudomonas fluorescens*	Ф = 8.0 mm MIC = 320 *μ*g/mL	
			*Salmonella abony*	Ф = 8.6 mm MIC = 320 *μ*g/mL	

Aerial part	Ethanolic extract	Agar-well diffusion method Broth microdilution method	*Staphylococcus aureus* 25923	Ф = 6 mm MIC = 6.25 mg/mL MBC = 12.5 mg/mL	[69]
			*Listeria monocytogenes* 4032	Ф = 8.1 ± 0.31 mm MIC = 6.25 mg/mL MBC = 12.5 mg/mL	
			*Bacillus cereus* ATCC 14579	Ф = 13.2 ± 0.23 mm MIC = <0.5 mg/mL MBC = 1 mg/mL	
			*Escherichia coli* ATCC 25929	Ф = 6 mm MIC = 25 mg/mL MBC = 50 mg/mL	
			*Pseudomonas aeruginosa* 195	Ф = 10 ± 0.043 mm MIC = 25 mg/mL MBC = 50 mg/mL	
			*Salmonella enterica*	Ф = 6 mm MIC = 12 mg/mL MBC = 25 mg/mL	

Aerial part	Essential oil	Disc diffusion assay	*Escherichia coli*	Ф = 13.66 ± 0.43 mm	[70]
			*Bacillus subtilis*	Ф = 26.21 ± 2.08 mm	
			*Mycobacterium smegmatis*	Ф = 28.34 ± 1.05 mm	

Leaves	Essential oil (2.7%)	Disc diffusion assay	*Escherichia coli*	Ф = 15.5 mm	[71]
			*Staphylococcus aureus*	Ф = 30.7 mm	
			*Acinetobacter baumannii*	Ф = 19 mm	
			*Bacillus cereus*	Ф = 16 mm	
			*Enterobacter cloacae*	Ф = 20 mm	

Flower	Essential oil (4.1%)	Disc diffusion assay	*Escherichia coli*	Ф = 20 mm	[71]
			*Staphylococcus aureus*	Ф = 45 mm	
			*Acinetobacter baumannii*	Ф = 35 mm	
			*Bacillus cereus*	Ф = 38 mm	
			*Enterobacter cloacae*	Ф = 22 mm	

Whole plant	Essential oil	Agar diffusion method Microdilution method	*Staphylococcus aureus*	Ф = 16 mm MIC = 1.1% (v/v)	[18]
			*Bacillus cereus*	Ф = 19 mm MIC = 1.1% (v/v)	
			*Escherichia coli*	Ф = 14.25 mm MIC = 1.25% (v/v)	

Flowering plant	Essential oil	Agar dilution method	*Escherichia coli* O157:H7	High antibacterial effect against the four pathogenic bacteria (MIC 0.05–0.4% (v/v))	[72]
			*Listeria monocytogenes* 2812 1/2a		
			*Salmonella typhimurium* SL 1344		
			*Staphylococcus aureus* ATCC 29213		

Flowering top	Essential oil	Agar diffusion method	*Pseudomonas aeruginosa* IH	Ф = 8 mm	[73]
			*Pseudomonas aeruginosa* CECT 110T	Ф = 8 mm	
			*Pseudomonas aeruginosa* CECT 118	Ф = 8 mm	
			*Pseudomonas Fluorescens* CECT 378	Ф = 11 mm	
			*Escherichia coli* k12	Ф = 13 mm	
			*Staphylococcus aureus* MBLA	Ф = 16 mm	
			*Staphylococcus aureus* CECT 976	Ф = 15 mm	
			*Staphylococcus aureus* CECT 794	Ф = 15 mm	
			*Bacillus subtilis* DCM 6633	Ф = 19 mm	
			*Bacillus capsulas*	Ф = 18 mm	
			*Enterococcus faecium* CECT 410	Ф = 13 mm	
			*Listeria innocua* CECT 4030	Ф = 21 mm	
			*Listeria monocytogenes* CECT 4032	Ф = 19 mm	

Aerial part	Essential oil (3.2%)	Microdilution assay	*Escherichia coli* 1 from patient	MIC = 0.33 mg/mL	[64]
			*Escherichia coli* ATCCS	MIC = 43 mg/mL	
			*Escherichia coli* 2 from patient	MIC = 0.36 mg/mL	
			*Escherichia coli* 1 from raw sheep milk	MIC = 0.51 mg/mL	
			*Escherichia coli* 2 from Raw Sheep Milk	MIC = 0.34 mg/mL	
			*Escherichia coli* 3 from raw sheep milk	MIC = 0.4 mg/mL	
			Enterohemorragic *Escherichia coli* (EHEC) O157	MIC = 0.21 mg/mL	
			Enteropathogenic *Escherichia coli* (EPEC)	MIC = 0.31 mg/mL	
			Enterotoxigenic *Escherichia coli* (ETEC)	MIC = 0.45 mg/mL	
			Enteroaggregative *Escherichia coli* (EAggEC)	MIC = 0.5 mg/mL	
			Enteroinvasive *Escherichia coli*	MIC = 0.7 mg/mL	

Leaves	Essential oil 1.35% (v/w)	Disc diffusion method	*Microbacterium testaceum*	High antibacterial effect (Ф > 20 mm) MIC = 0.025% (v/v) MBC = 0.033% (v/v)	[63]
			*Serratia marcescens*	Important antibacterial activity (15 < Ф < 19 mm) MIC = 0.033% (v/v) MBC = 0.05% (v/v)	

Leaves	Aqueous extract 100 mg/mL (w/v)	Disc diffusion method	*Microbacterium testaceum*	Slight antibacterial activity (Ф < 8 mm)	[63]
			*Serratia marcescens*	Slight antibacterial activity (Ф < 8 mm)	

Aerial parts	Essential oil	Microdilution method	*Mycobacterium aurum* A+	MIC = 0.015% (v/v) MBC = 0.015% (v/v)	[16]
			*Mycobacterium smegmatis* mc2-155	MIC = 0.062% (v/v) MBC = 0.062% (v/v)	

**Table 4:** Antioxidant effects of *T. satureioides*.

Parts used	Extracts	Methods used	Findings	Reference
Aerial parts	Crude extract	DPPH free radical scavenging activity assay	IC_50_ = 0.44 ± 0.06 mg/mL	[39]
		FRAP assay	IC_50_ = 41.41 ± 4.55 mmol/L	
	Ethyl acetate extract	DPPH assay	IC_50_ = 0.33 ± 0.02 mg/mL	
		FRAP assay	IC_50_ = 82.69 ± 2.29 mmol/L	
	Methanolic extract	DPPH assay	IC_50_ = 0.71 ± 0.09 mg/mL	
		FRAP assay	IC_50_ = 55.99 ± 2.21 mmol/L	
	Aqueous extract	DPPH assay	IC_50_ = 0.85 ± 0.06 mg/mL	
		FRAP assay	IC_50_ = 25.46 ± 2.71 mmol/L	
	Dichloromethane extract	DPPH assay	IC_50_ = 0.48 ± 0.05 mg/mL	
		FRAP assay	IC_50_ = 33.48 ± 0.08 mmol/L	
Aerial parts	Aqueous extract	TBARS method	Significant inhibition of lipid peroxidation product (MDA)	[24]
		DPPH assay	IC_50_ = 0.44 ± 0.01 mg/mL	
		FRAP assay	IC_50_ = 40.14 ± 4.55 mmol/g	
Aerial parts	Aqueous extract	ABTS assay	IC_50_ = 14.65 ± 0.36 *μ*g/mL	[76]
Aerial part	Essential oil (1.86%)	DPPH assay	IC_50_ = 167.00 ± 2.47 *μ*g/mL	[15]
		Reducing power technique	IC_50_ = 176.89 ± 1.02 *μ*g/mL	
Aerial parts	Essential oil (2%)	DPPH assay	IC_50_ = 0.21 ± 1.17 mg/mL	[49]
		Reducing power assay	IC_50_ = 0.23 ± 0.67 mg/mL	
		*β*-Carotene/linoleic acid bleaching assay	IC_50_ = 0.21 ± 1.74 mg/mL	
		ABTS assay	IC_50_ = 0.15 ± 0.36 mg/mL	
Flowering top	Essential oil	Reducing power assay	Absorbance = 0.507 ± 0.019	[73]
		DPPH assay	Percent inhibition = 42.99%	
		*β*-Carotene test	Percent inhibition = 74.50%	
Aerial part	Ethyl acetate extract	DPPH assay	IC_50_ = 109.98 ± 3 *μ*g/mL	[60]
Aerial part	Essential oil	Reducing power assay	Percent inhibition = 24.64 ± 0.03%	[47]
		TEAC assay	TEAC = 159.00 ± 0.01 mMof Trolox/m	
		*β*-Carotene bleaching assay	Percent inhibition = 83.87 ± 0.10%	
		DFRS assay	Percent inhibition = 68.55 ± 0.01%	
Aerial part	Essential oil	Ferric reducing capacity	EC_50_ = 177.13 ± 2.1 mg/mL	[46]
		DPPH assay	IC_50_ = 122.53 ± 2.38 *μ*g/mL	
Leaves	Methanolic extract	Free radical scavenging activity (DPPH° test)	SC_50_ = 14.6 *μ*g	[14]
Aerial parts	Essential oil	DPPH assay	IC_50_ = 0.25 ± 0.03 mg/mL	[43]
		*β*-Carotene/linoleic acid assay	Percent inhibition = 81.78 ± 0.37%	
		TBARS assay	I_50_= 300.32 ± 1.50 mg/mL	
Aerial part	Aqueous extract	FRAP assay	IC_50_ = 50.79 ± 2.02 mmol Trolox/g	[13]
		Malondialdehyde (MDA) assay	Important antioxidant activity	
		DPPH assay	IC_50_ = 0.480 ± 0.010 mg/mL	
		AAPH-induced oxidative erythrocyte hemolysis assay	Neutralization of the free radicals liberated by the AAPH	
Aerial part	Essential oil	Reducing power	IC_50_ = 85.47 ± 0.95 *μ*g/mL	[21]
		*β*-Carotene/linoleic acid assay	IC_50_ = 64.26 ± 0.70 *μ*g/mL	
		DPPH	IC_50_ = 108.15 ± 1.54 *μ*g/mL	
Aerial part	Essential oil	DPPH assay	IC_50_ = 0.81 mg/mL	[44]
Aerial part	Hot water extract	DPPH assay	IC_50_ = 15.99 ± 0.47 *μ*g/mL	[77]
		Reducing power assay	IC_50_ = 20.33 ± 0.19 *μ*g/mL	
		*β*-Carotene bleaching	IC_50_ = 14.69 ± 0.69 *μ*g/mL	
	Cold water extract	DPPH assay	IC_50_ = 53.42 ± 1.17 *μ*g/mL	
		Reducing power assay	IC_50_ = 64.32 ± 0.52 *μ*g/mL	
		*β*-Carotene bleaching	IC_50_ = 50.20 ± 0.33 *μ*g/mL	
	Methanol extract	DPPH assay	IC_50_ = 30.24 ± 0.19 *μ*g/mL	
		Reducing power assay	IC_50_ = 30.48 ± 0.52 *μ*g/mL	
		*β*-Carotene bleaching test	IC_50_ = 86.38 ± 0.85 *μ*g/mL	
Aerial part	Hexane extract	DPPH assay	IC_50_ = 275.71 ± 11.26 *μ*g/mL	[22]
		ABTS assay	IC_50_ = 127.38 ± 3.83 *μ*g/mL	
		FRAP assay	97.819 ± 0.377 mg equivalent ascorbic acid/g of extract	
	Dichloromethane extract	DPPH assay	IC_50_ = 8.18 ± 0.07 *μ*g/mL	
		ABTS assay	IC_50_ = 80.09 ± 0.65 *μ*g/mL	
		FRAP assay	153.457 ± 0.247 mg equivalent ascorbic acid/g of extract	
	Ethyl acetate extract	DPPH assay	IC_50_ = 23.75 ± 0.67 *μ*g/mL	
		ABTS assay	IC_50_ = 85.16 ± 3.22 *μ*g/mL	
		FRAP assay	123.004 ± 0.377 mg equivalent ascorbic acid/g of extract	
	Water-ethanol extract	DPPH assay	IC_50_ = 3.86 ± 0.07 *μ*g/mL	
		ABTS assay	IC_50_ = 51.27 ± 0.82 *μ*g/mL	
		FRAP assay	233.292 ± 0.377 mg equivalent ascorbic acid/g of extract	
Leaves	Hot aqueous extract	DPPH assay	IC_50_ = 0.343 ± 0.011 mg/mL	[69]
		Iron-ferrous chelating power assay	IC_50_ = 0.4539 ± 0.011 mg/mL	
	Cold aqueous extract	DPPH assay	IC_50_ = 0.652 ± 0.013 mg/mL	
		Iron-ferrous chelating power assay	IC_50_ = 0.6394 ± 0.014 mg/mL	
	Ethanolic extract	DPPH assay	IC_50_ = 0.247 ± 0.011 mg/mL	
		Iron-ferrous chelating power assay	IC_50_ = 0.3341 ± 0.012 mg/mL	
Leaves	Methanolic extract	DPPH assay	Percent inhibition = 92.24%	[75]

## Data Availability

All data analyzed during this investigation are available from the corresponding author.

## Abbreviations

EOs: Essential oils
HPLC: High-performance liquid chromatography
^1^H NMR: Proton nuclear magnetic resonance
GC-FID: Gas chromatograph-flame ionization detection
GC-MS: Gas chromatography-mass spectrometry
MIC: Minimal inhibitory concentration
MBC: Minimum bactericidal concentration
Ф: Inhibition zone diameter
ABTS: 2,2′-Azino-bis 3-ethylbenzothiazoline-6-sulphonic acid
FRAP: Ferric reducing antioxidant power
DPPH: 2,2-Diphenyl-1-picrylhydrazyl
TBARS: Thiobarbituric acid reactive substances
APPH: 2,2-Azobis 2-amidinopropane dihydrochloride
BHT: Butylated hydroxytoluene
MFC: Minimal fungicidal concentration
MTT: 3-(4,5-Di-methylthiazol-2-yl)-2,5-diphenyl tetrazolium bromide
TAE: Tannic acid equivalent.
